# Optimization of Online Moisture Prediction Model for Paddy in Low-Temperature Circulating Heat Pump Drying System with Artificial Neural Network

**DOI:** 10.3390/s25072308

**Published:** 2025-04-05

**Authors:** Yi Zuo, Abdulaziz Nuhu Jibril, Jianchun Yan, Yu Xia, Ruiqiang Liu, Kunjie Chen

**Affiliations:** College of Engineering, Nanjing Agricultural University, Nanjing 210031, China; 2021212014@stu.njau.edu.cn (Y.Z.); 2021212026@stu.njau.edu.cn (A.N.J.); 2022212005@stu.njau.edu.cn (J.Y.); 2023112032@stu.njau.edu.cn (Y.X.); liuruiqiang@stu.njau.edu.cn (R.L.)

**Keywords:** heat pump dryer, paddy drying, artificial neural network, model optimization

## Abstract

The accurate prediction of moisture content is crucial for controlling the drying process of agricultural products. While existing studies on drying models often rely on laboratory-scale experiments with limited data, real-time and high-frequency data collection under industrial conditions remains underexplored. This study collected and constructed a multi-dimensional dataset using an industrial-grade data acquisition system specifically designed for heat pump low-temperature circulating dryers. An artificial neural network (ANN) prediction model for moisture content during the rice drying process was developed. To prevent model overfitting, K-fold cross-validation was utilized. The model’s performance was evaluated using the mean squared error (MSE) and the coefficient of determination (R^2^), which also helped determine the preliminary structure of the ANN model. Bayesian regularization (trainbr) was then employed to train the network. Furthermore, optimization was conducted using neural network weights (RI) analysis and Sobol variance contribution analysis of the input variables to simplify the model structure and improve predictive performance. The experimental results showed that optimizing the model through RI sensitivity analysis simplified its topology to a 5-14-1 structure. The optimized model exhibited not only simplicity but also high prediction accuracy, achieving R^2^ values of 0.969 and 0.966 for the training and testing sets, respectively, with MSEs of 5.6 × 10^−3^ and 6.3 × 10^−3^. Additionally, the residual errors followed a normal distribution, indicating that the predictions were reliable and realistic. Statistical tests such as *t*-tests, F-tests, and Kolmogorov–Smirnov tests revealed no significant differences between the predicted and actual values of rice moisture content, confirming the high consistency between them.

## 1. Introduction

Drying is a critical step in grain production, and the use of dryers is essential for reducing losses and ensuring food security [1]. Currently, commonly used grain dryers include counterflow dryers [2], crossflow dryers [3], mixed-flow dryers [4], and low-temperature cyclic dryers. Among these, low-temperature cyclic dryers are widely employed in southern China, Japan, and Southeast Asia for drying rice [5].

Modeling and monitoring changes in moisture content during the drying process are vital for formulating drying strategies, controlling the drying process, and managing drying quality and costs. Although some existing grain dryers are equipped with online moisture content detection systems, grain drying is a process with high inertia. Adjusting drying parameters based on real-time moisture detection often leads to significant delays and overshoots. Thus, modeling the changes in grain moisture content during drying to predict such changes in advance has significant practical implications. Historically, researchers have primarily relied on empirical or physical models to describe the drying process, developing classical models such as the Page model and diffusion models. However, these models are usually derived under controlled laboratory conditions, considering only two variables: drying temperature and humidity. As such, they deviate significantly from complex real-world drying processes and are unsuitable for real-time monitoring or control of actual drying operations [6]. Moreover, physical models require solving complex mass and heat transfer equations and making numerous simplifying assumptions about the equipment and material properties, which significantly reduces their prediction accuracy [7]. Therefore, accurately predicting changes in rice moisture content during novel heat pump low-temperature cyclic drying processes remains a considerable challenge.

Artificial neural networks (ANNs) represent an innovative approach to data analysis and modeling. They can establish relationships between inputs, such as drying characteristic parameters, and outputs, such as moisture content, using experimental data without requiring an understanding of their intrinsic physical relationships. Currently, numerous studies have demonstrated the feasibility of using ANNs to model the drying processes of various agricultural products. Nanvakenari et al. [8] conducted a laboratory-scale fluidized bed drying (FBD) experiment on paddy, processing approximately 300 g of samples per trial under predetermined temperature and fluidization velocity conditions. An artificial neural network (ANN) combined with response surface methodology (RSM) was employed to develop a predictive model for the relationship between drying time and quality attributes, including paddy yield, whiteness index, water absorption ratio, and elongation ratio. In this study, temperature and fluidization velocity were treated as constant parameters during the drying process, and moisture data were collected only at the end of drying. Jibril et al. [9] investigated the performance of various machine learning algorithms in predicting the moisture content of corn under different drying temperature conditions. Each drying test used 25 kg of corn and moisture was manually measured every 30 min. The results indicated that the support vector machine (SVM) model exhibited the best predictive performance in simulating the corn drying process. Qadri et al. [10] applied machine learning to simulate the microwave drying of papaya, demonstrating that SVR outperformed other models in predicting drying time. Chokphoemphun et al. [11] conducted experimental research on paddy drying using a tower-type fluidized bed dryer, with each batch processing 800 g of samples. Moisture content was manually measured by weighing every 10 min, resulting in a dataset of 176 samples. Based on this dataset, an artificial neural network (ANN) model was developed for moisture prediction. In the model, chamber configuration and airflow velocity were treated as fixed input parameters, without considering parameter fluctuations during the drying process. The study optimized model performance by adjusting activation functions, network structure, sampling method, and the number of training epochs. Liu et al. [12] proposed using an ANN to predict energy and energy use parameters during the hot air impingement drying of mushroom slices, concluding that ANN models with specific training algorithms and transfer functions can effectively predict the performance of such drying systems. Beigi et al. [13] compared the performance of ANNs in predicting the moisture content of thin layer rice drying under varying air temperatures and flow rates against nine mathematical models. Their findings indicated that an ANN with a 4-18-18-1 topology and a Levenberg Marquardt back propagation training algorithm yielded the highest correlation coefficient and the lowest mean square error. Marić et al. [14] applied an ANN to predict the physical and chemical properties of root vegetables following conventional drying, achieving a coefficient of determination (R^2^) exceeding 80%. Similarly, Huang et al. [15] conducted drying experiments on apple slices using a laboratory-scale oven, with each batch processing approximately 100 g of samples. They compared the performance of deep neural networks (DNNs), multilayer perceptrons (MLPs), and support vector regression (SVR) in predicting the moisture content of apple slices. The results showed that the DNN model outperformed the others, achieving the highest coefficient of determination (R^2^) and the lowest mean absolute error (MAE). Kaveh et al. [16] used adaptive neuro-fuzzy inference systems (ANFISs) and ANNs to predict the drying characteristics of cantaloupe, potatoes, and garlic in convection hot air dryers. In each experiment, the individual sample mass was approximately 40 g. Collectively, these studies confirm the feasibility of using ANNs to predict the drying characteristics of agricultural products. However, most of these studies were based on laboratory-scale small-batch drying experiments, where the amount of material processed per run was relatively small, and drying parameters such as temperature, air velocity, and initial moisture content were well controlled. As a result, the developed models have limited applicability in complex and dynamically changing industrial-scale drying environments [17]. Moreover, data in these studies were mostly collected manually, with limited parameter dimensions and low sampling frequency, making it difficult to comprehensively capture the real-time dynamics of the drying process and thereby limiting the practical value of the models.

Modeling the drying processes of grains in actual production environments poses several challenges: (1) In industrial drying processes, the moisture content of mechanically harvested paddy typically ranges from 20% to 24%, exhibiting significant non-uniformity. Meanwhile, the hot air temperature in large-scale drying equipment can fluctuate by up to 30 °C during operation, further contributing to the uneven distribution of moisture and temperature among the materials. This results in pronounced coupling effects during the drying process, thereby increasing the complexity of both modeling and process control. [18]. (2) The drying environment in actual grain production is complex and lacks the ideal conditions found in laboratory-scale experiments, making it highly susceptible to disturbances from heat sources, ambient temperature and humidity, and equipment operating conditions [19]. Zhang et al. [20] proposed replacing traditional MPC optimization with LSTM neural networks and developed an industrial-scale multi-stage countercurrent drying model for rice. Their approach improved online prediction accuracy and response speed. Jin et al. [21] designed a BPNN neural network with a 13-24-1 topology to predict rice moisture in continuous dryers, using inputs such as ambient temperature and humidity as well as exhaust temperature and humidity from three drying sections, totaling 13 characteristic parameters. Their results showed that the model had small steady state errors and excellent anti-interference capabilities (R^2^ = 0.8842). Dai et al. [22] experimentally investigated the infrared radiation and convection (IRC) drying process of grains. Drying parameters, including drying time, initial moisture content, initial grain temperature, grain temperature during infrared and convection stages, post-drying grain temperature, hot air temperature, and grain discharge speed, were used as inputs for a BPNN model to predict the moisture content and drying rate at the outlet of the IRC grain dryer. The findings validated BPNN as an effective tool for characterizing and controlling the IRC grain drying process. Li et al. [23] proposed a countercurrent grain dryer moisture prediction and control scheme based on neural networks. Using experimental data—including initial moisture content, grain temperature, hot air temperature, hot air humidity, and grain discharge rate—they trained a nonlinear autoregressive neural network to develop a rice moisture prediction model. This method accounted for time-dependent memory effects and effectively addressed the nonlinear nature of moisture content.

Although the above studies provide valuable references and foundational insights into predicting moisture content during the drying processes of industrial-scale dryers using ANNs, several limitations remain: (1) many experiments were conducted in laboratory environments with manually collected data, resulting in small datasets and incomplete drying information that cannot fully represent real-world drying conditions; (2) most models focused solely on the relationship between dryer process parameters and moisture content, without considering the characteristics of heat source equipment. Notably, there is a lack of research on low-temperature circulating dryers, which are the predominant type in China, as well as on heat pump systems. In addition, the types of drying equipment involved in existing industrial-scale applications—such as continuous dryers and infrared–convection combined drying systems—exhibit fundamentally different drying mechanisms compared to the low-temperature circulating heat pump dryer employed in this study. To address these gaps, this study focuses on a heat pump-based low-temperature circulating dryer and proposes an ANN-based rice moisture prediction model. The primary contributions and innovations of this study are as follows: (1) To support the development of industrial-scale dryer moisture prediction models, an online data acquisition system was developed to collect multi-dimensional drying characteristic data in real time during the rice drying process in a heat pump-based low-temperature circulating dryer. These data include active power, heat pump energy consumption, ambient temperature, drying temperature, drying humidity, exhaust temperature, exhaust humidity, grain temperature, and moisture content, with a data collection frequency as high as one-second intervals. This system addresses the challenges of subjective manual data collection and limited data quantity, enabling datasets that accurately reflect actual drying processes. (2) A heat pump-based low-temperature circulating dryer moisture prediction ANN model was proposed. For the first time, heat pump and low-temperature circulating dryer characteristic data were integrated to predict rice moisture. Various algorithms and topological structures were employed to optimize the neural network. By applying RI analysis and Sobol sensitivity analysis, the influence of different drying characteristics on moisture content was investigated from the perspectives of input weight coefficients and nonlinear correlations among inputs. This approach optimized the network structure while preserving characteristic details, thereby enhancing prediction accuracy and simplifying the model structure.

## 2. Materials and Methods

### 2.1. Working Principle of the Low-Temperature Circulation Heat Pump Dryer

**Figure 1:**
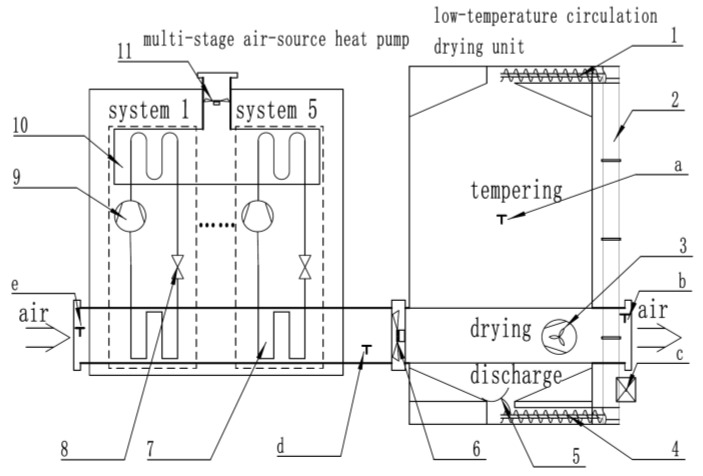
Schematic diagram of the low-temperature heat pump circulating dryer: 1—upper screw conveyor; 2—lifting motor; 3—dust removal machine; 4—lower screw conveyor; 5—grain discharge wheel; 6—exhaust fan; 7—condenser; 8—expansion valve; 9—compressor; 10—evaporator; 11—evaporator fan; a—grain temperature sensor; b—exhaust temperature and humidity sensor; c—moisture analyzer; d—drying temperature and humidity sensor; e—ambient temperature sensor.

The heat pump dryer consists of a multi-stage air-source heat pump and a low-temperature circulation drying unit. The structure is illustrated in Figure 1. The low-temperature circulation unit used is the CPR-165 (Suncue Company Ltd., Taichung, China), with dimensions of 4287 × 2745 × 9671 mm (L × W × H). Its internal structure includes a tempering chamber, drying chamber, and discharge chamber, capable of drying between 6500 and 13,250 kg of paddy per batch. The drying process operates as follows: Paddy is first transported into the dryer through an elevator hopper and upper screw conveyor, then flows sequentially from top to bottom through the tempering section, drying section, and discharging section. In the drying section, the paddy is dried in a layered manner, flowing downward through the section in thin layers. The hot air passes through the paddy layers to achieve uniform moisture removal, and the moisture reduction rate can be controlled by adjusting the hot air temperature and velocity. When the paddy reaches the discharging section, its moisture content is measured using an online moisture sensor. If the target moisture level has not been reached, the grain is recirculated via the discharging wheel, lower screw conveyor, elevator, and upper screw conveyor back into the dryer for another drying cycle. After multiple cycles, once the desired final moisture content is achieved, the paddy is discharged from the dryer. Drying air generated by the heat pump is delivered to the drying chamber via an exhaust fan, where it exchanges heat with the grains, removing excess moisture. The humid air is then expelled through the exhaust outlet. The multi-stage air-source heat pump, developed by our laboratory, consists of five interconnected systems (the dotted line box in the figure is one system). Each system includes key components such as compressors, condensers, evaporators, and expansion valves. Ambient air passes through the condenser in stages, increasing in temperature. By activating different numbers of heat pump systems, the temperature of the drying air can be precisely controlled.

### 2.2. Drying Characteristic Data Collection

#### 2.2.1. Automatic Data Collection System

**Figure 2:**
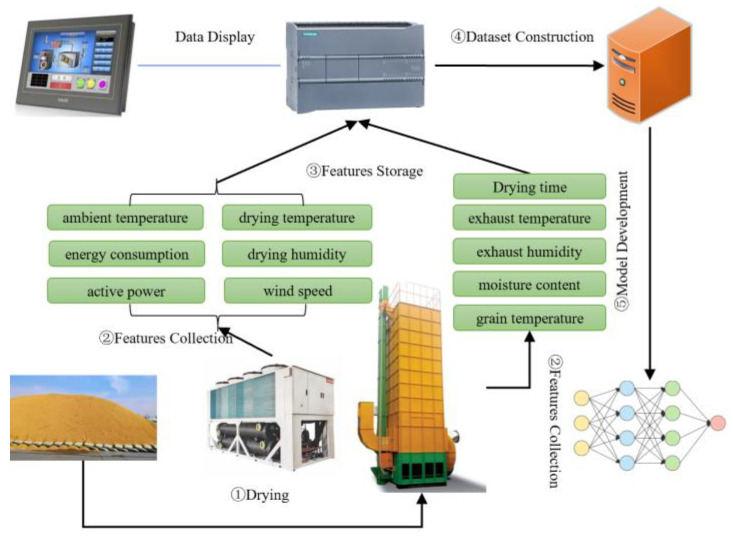
Automatic data collection system for drying characteristics.

To meet the development requirements of an industrial-grade dryer moisture prediction model, it is essential to collect as many multidimensional characteristic data points as possible during the drying process. Therefore, this study proposes an automated drying characteristic data collection system capable of real-time data acquisition and storage throughout the drying process. Upon completion of drying, the constructed dataset is transmitted to a computer for model development. The automated drying characteristic data collection system is illustrated in Figure 2. Essential sensors are installed on the multi-stage air-source heat pump and low-temperature circulation dryer to capture drying characteristics. A programmable controller is configured to set the data acquisition frequency and to receive and store the characteristic data transmitted by the sensors via signal lines. The specifications of the sensors are provided in Table 1. Key parameters, including temperature, humidity, airflow velocity, and moisture content, directly influence the accuracy of the prediction model. Given that these parameters undergo continuous changes influenced by multiple factors during the drying process, high-precision sensors are necessary. Active power and active energy represent real-time power consumption and total energy consumption during the drying process, respectively. These measurements are collected using power meters installed on the power lines of the multi-stage air-source heat pump. The locations of the sensor installations are depicted in Figure 1.

#### 2.2.2. Experiment and Data Preprocessing

Between 15 October and 15 November 2022, paddy drying experiments were conducted at the Hetian Agricultural Machinery Cooperative in Gaochun, Nanjing, China. To ensure the experimental conditions aligned with real industrial drying processes, freshly harvested Nanjing 46 paddy was collected from nearby fields, cleaned of impurities, and dried immediately. Each drying experiment used 10,000 kg of paddy. During the experiments, the ambient temperature ranged from 12 °C to 24 °C, drying temperatures were maintained between 45 °C and 63 °C, airflow velocities ranged from 7 to 13 m/s, initial moisture content varied from 22% to 28%, and the final moisture content was standardized to 14%. A total of four drying experiments was conducted. Prior to the experiments, the moisture content was measured using the 105 °C oven method, and the moisture meter was calibrated. During the experiments, data were collected every 10 s, including time, moisture content, ambient temperature, drying temperature, drying humidity, exhaust temperature, exhaust humidity, grain temperature, air velocity, active power, and active energy consumption. The outliers were removed, and the data collected within each minute were averaged. This resulted in the creation of a drying characteristic dataset consisting of 4103 data points. Among these, 10% of the data were randomly selected as the test set, while the remaining 90% were used for model training and five-fold cross-validation.

### 2.3. Artificial Neural Network Modeling

#### 2.3.1. Neural Network Architecture

**Figure 3:**
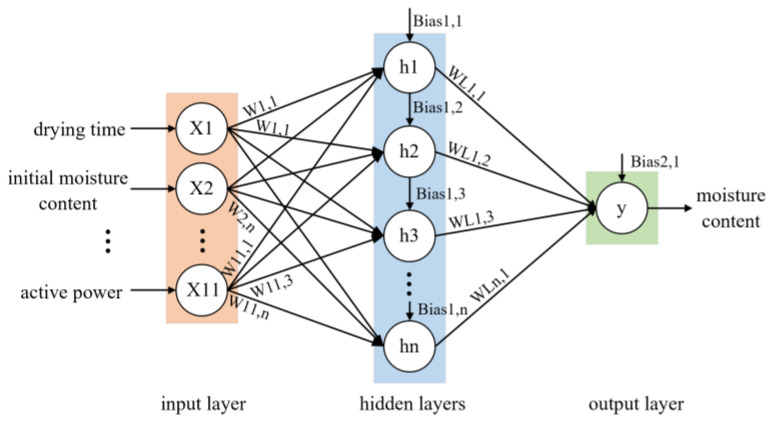
Neural network structure for moisture prediction.

An artificial neural network was employed as a data-driven approach to predict the moisture variation during the drying process of paddy, aiming to capture complex nonlinear relationships among drying parameters that are difficult to express analytically in industrial settings. The idea was to find a design that would produce the fewest differences between the estimated and experimental moisture content. The significance of choosing optimal ANN designs to accurately forecast the moisture content as an output given particular input factors was highlighted [9]. The neural network consists of an input layer, multiple hidden layers, and an output layer. In this study, the input layer consists of 11 neurons representing the following variables: drying time, initial moisture content, ambient temperature, drying temperature, drying humidity, exhaust temperature, exhaust humidity, grain temperature, air velocity, active power, and active energy consumption. The output of the network is the paddy moisture content. These input variables all influence moisture content, making them suitable for predicting the moisture content. To ensure a simple network structure, only one hidden layer was considered in this study. The number of neurons in the hidden layer was optimized based on the performance of the network during training. The network structure is shown in Figure 3.

The activation function which determines the neuron’s output is used as the input of the total buried layer neurons’ input, weights, and biases. The prediction error in which the activation function’s derivative can scale is used to assess the network’s performance after the output has been calculated. The training procedure modifies the network’s weights and biases, enabling the model to learn from the data and generate precise predictions. The weights and biases are changed during backpropagation if the error target does not reach the predetermined maximum. The training set aims to define the optimal weights and biases that minimize the output error.

#### 2.3.2. Training and Optimization Methods

The best training function was selected based on several factors, such as the sample size of the training dataset, the complexity of the network, and the target error. Several representative training functions were chosen, including Bayesian Regularization BP (br), Levenberg–Marquardt BP (lm), RPROP BP (rp), BFGS quasi-Newton BP (bfg), gradient descent with momentum and adaptive learning rate BP (gdx), and scaled conjugate gradient BP (scg). The best training function for processing drying data was identified using random order weights/biases (R) and sequential order weights/biases (S) with a logistic sigmoid activation function in the hidden layer and a linear activation function in the output layer. Hidden layers with different neuron counts ranging from 1 to 20 were tested.

The performance of different activation functions in Table 2 was evaluated after determining the best training function which includes sigmoid (logsig), hyperbolic tangent sigmoid (tansig), Elliot sigmoid (elliotsig), positive linear (poselin), radial basis (radbas), and triangular basis (tribas). This was performed to determine the optimal activation function and number of neurons for the hidden layer. However, the activation function of the output layer was linear for all the training sets.

### 2.4. Feature Parameter Contribution Analysis

#### 2.4.1. RI Analysis

The relative impact (RI) of input parameters was determined based on the weight coefficients of the developed ANN model, and the output variables were calculated using the global sensitivity equation described by Yoon [24]:

(1) $$RI_{ij}(\%)=\frac{\sum_{k=0}^{n}\left(w_{ik}\cdot w_{kj}\right)}{\sum_{i=0}^{m}\left|\sum_{k=0}^{n}\left(w_{ik}\cdot w_{kj}\right)\right|}\cdot 100\%$$

where w—weight coefficient in the ANN model, i—input variable, j—output variable, k—hidden neuron, n—number of hidden neurons, and m—number of inputs. This method calculates the relative contribution of each input variable to the output based on the weight values of the neural network model. The absolute value of the weights is used to measure the importance of each input variable.

#### 2.4.2. Sobol Sensitivity Analysis

Sobol sensitivity analysis is based on the contribution of input variables to the variance of the output variable and is capable of capturing the nonlinear interaction effects between input variables [25]. This method takes into account the influence of variations in input variables across the entire input space on the output, not just relying on weights.

For a model $Y=f(X_{1}\;,X_{2}\;,\ldots ,X_{d}\;)$, where *X_i_* is the input variable, the output *Y* can be represented as:

(2) $$Y=E[Y]+\sum_{i=1}^{d}\left(X_{i}-E[X_{i}]\right)f_{i}(X_{i})+\sum_{i<j}\left(X_{i}-E[X_{i}]\right)\left(X_{j}-E[X_{j}]\right)f_{ij}(X_{i},X_{j})+\cdots$$

where *f_i_* is a function of a single input variable, *f_ij_* represents the interaction function of two input variables, and so on.

Sobol sensitivity analysis calculates the sensitivity indices of input variables through variance decomposition. The total variance *V(Y)* is decomposed into contributions from each input variable and their interactions:

(3) $$V(Y)=\mathrm{Var}(Y)=\mathrm{Var}\left[E[Y|X_{1},\ldots ,X_{d}]\right]+\sum_{i=1}^{d}\mathrm{Var}\left[E[Y|X_{i}]\right]+\sum_{i<j}\mathrm{Var}\left[E[Y|X_{i},X_{j}]\right]+\cdots$$

The influence of a single input variable *X_i_* on the model output is computed using the First-order Sobol Index (*S_i_*):

(4) $$S_{i}=\frac{V\left(E[Y|X_{i}]\right)}{V(Y)}$$

The effect of interactions between *X_i_* and other input variables on the model output is represented by the Total-order Sobol Index (*ST_i_*):

(5) $$ST_{i}=\frac{\mathrm{Var}\left[E[Y|X_{i}]\right]+\sum_{j\neq i}\mathrm{Var}\left[E[Y|X_{i},X_{j}]\right]+\cdots }{V(Y)}$$

### 2.5. Normalization and K-Fold Cross-Validation

The dataset used in this study has features with significantly varying ranges. We applied Z-score normalization to scale all input features to a similar range, ensuring more efficient and stable updates during gradient descent, and preventing issues like gradient vanishing or explosion. Normalization also enhances the model’s generalization ability and reduces the risk of overfitting [26]. Z-score normalization is defined as:

(6) $$x^{\prime }=\frac{x-\mu }{\sigma }$$

where x is the original data, $x^{\prime }$ is the normalized data, μ is the mean of the data, and σ is the standard deviation of the data.

Overfitting is a common challenge in neural network training. Cross-validation helps mitigate overfitting by evaluating model performance on independent datasets and selecting optimal parameters. In this study, a K-fold cross-validation method was employed. The dataset was divided into k folds, with each iteration using k-1 folds for training and the remaining fold for validation. By averaging the errors across multiple training runs, this method provides a more accurate evaluation of model performance and reduces the likelihood of overfitting. Specifically, the dataset of 4103 entries was shuffled, with 10% randomly selected as the test set. The remaining data were used for training the neural network through five-fold cross-validation.

### 2.6. Model Performance Evaluation

The model’s performance and effectiveness were assessed using the following criteria: coefficient of determination (R^2^) and mean square error (MSE). These criteria provide a comprehensive evaluation of the model’s accuracy, precision, and overall predictive power. By considering multiple performance metrics, the assessment ensures a thorough understanding of the model’s capabilities and limitations.

(7) MSE=∑i=1n(yi−y^i)2n

(8) R2=1−∑i=1n(yi−y^i)2∑i=1n(y^i−μ)2

### 2.7. Comparison of Mathematical Models

Table 3 shows the equations used to determine the best model for explaining the paddy drying curve. Five semi-empirical drying equations were operated to describe the paddy drying kinetics in this investigation.

## 3. Results and Discussion

### 3.1. Selection of the Training Function

**Figure 4:**
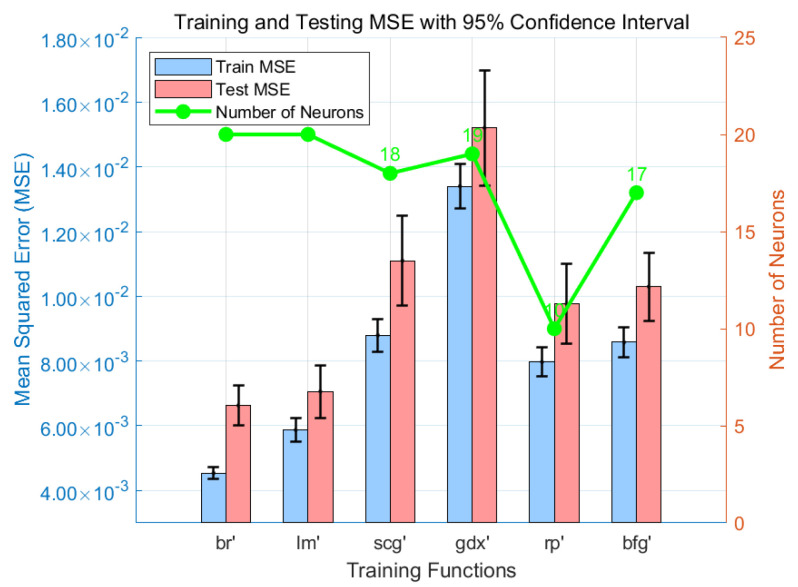
Test set errors and required number of neurons for different training functions.

The log-sigmoid (logsig) function was used as the hidden layer transfer function, whereas the positive linear (poslin) function was employed to transmit the output layer. Various training functions were tested to determine the optimal number of hidden neurons that achieve minimal MSE. The training results are shown in Figure 4. Bayesian Regularization BP (br) had the lowest MSE on the test set (MSE = 6.6 × 10^−3^, R^2^ = 0.962), followed by Levenberg–Marquardt BP (lm) (MSE = 7.0 × 10^−3^, R^2^ = 0.959). The minimum number of neurons (NN) needed to reach the target accuracy for Levenberg–Marquardt BP (lm), Bayesian Regularization BP (br), BFGS quasi-Newton BP (bfg), scaled conjugate gradient BP (scg), RPROP BP (rp), and gradient descent with momentum and adaptive learning rate BP (gdx) were 20, 20, 17, 18, 10, and 19, respectively. This indicates that training the network with br and lm requires a more complex structure to achieve minimal MSE. However, Levenberg–Marquardt BP (lm) and Bayesian Regularization BP (br) outperform other training functions due to their relatively complex structures. Therefore, Bayesian Regularization BP (br) was chosen as the optimal training function for this network as it exhibited superior performance. This revealed that a single hidden layer provides a highly accurate model.

To further evaluate the stability and generalization ability of the model under different training functions, 95% confidence intervals were used to visualize the MSEs of the training and testing sets. As shown in Figure 4, Bayesian Regularization BP (br) and Levenberg–Marquardt BP (lm) not only achieved the lowest mean errors, but also exhibited significantly narrower confidence intervals, indicating low prediction variability and good stability. In contrast, training functions such as gdx and scg, though acceptable in certain cases, showed wider confidence intervals on the test set, suggesting less stable prediction performance and limited generalization. Additionally, the rp function achieved moderate mean errors with fewer neurons and smaller error fluctuations, indicating a relatively simple yet stable network structure. Overall, the analysis of error metrics and confidence intervals further confirms the superiority of the br training function in this study.

### 3.2. Selection of the Activation Function

**Figure 5:**
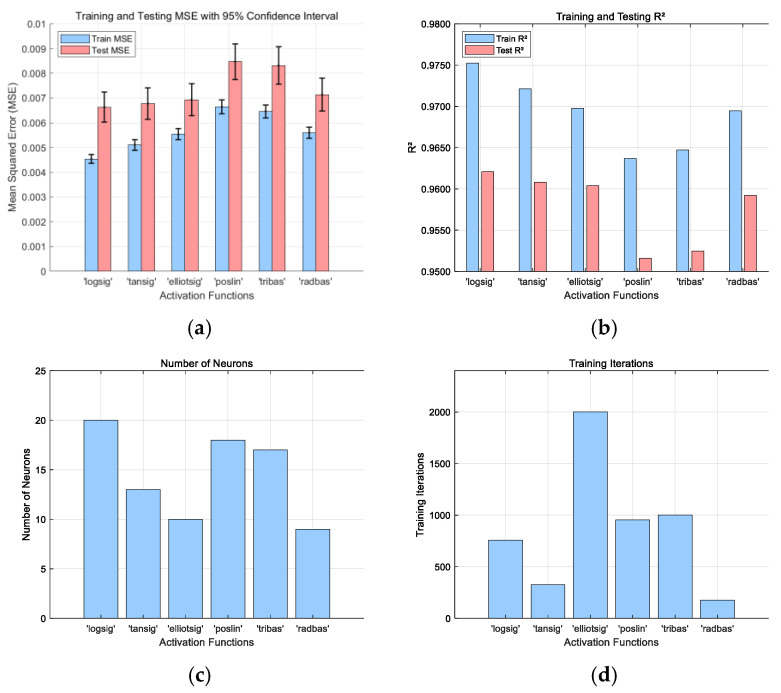
Activation function optimization results: (**a**) minimum MSE, (**b**) maximum R^2^, (**c**) number of hidden layer neurons for optimal performance, and (**d**) number of epochs for optimal performance.

The performance of various activation functions was tested using the Bayesian Regularization BP (br) function for training, with the number of neurons ranging from 1 to 20, and a positive linear (poslin) transfer function in the output layer. The activation functions comprised sigmoid-based functions—log-sigmoid (logsig), hyperbolic tangent sigmoid (tansig), and Elliot symmetric (elliotsig), as well as positive linear (poslin), radial (radbas), and triangular (tribas). The training results are shown in Figure 5. The log-sigmoid (logsig) function achieved optimal results at 759 epochs, with training and testing MSE values of 4.5 × 10^−3^ and 6.6 × 10^−3^ and R^2^ values of 0.975 and 0.962, respectively. Consequently, the Bayesian Regularization BP (br) training method with a log-sigmoid (logsig) activation function and 20 hidden neurons was chosen as the optimal network structure.

**Figure 6:**
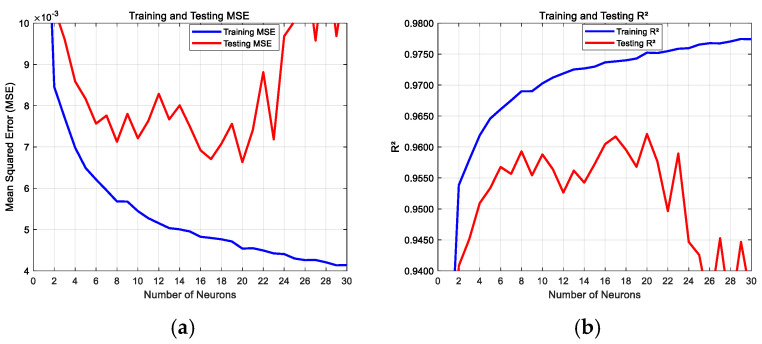
Training process with different numbers of hidden layer neurons: (**a**) training and testing MSE; (**b**) training and testing R^2^.

The log-sigmoid (logsig) activation function in the hidden layer achieved MSE and R^2^ values for different numbers of hidden neurons, as shown in Figure 6. With the increase in the number of neurons, the training set consistently dropped to MSE = 4.5 × 10^−3^ and R^2^ = 0.975, while the test set reached a minimum of MSE = 6.6 × 10^−3^ and R^2^ = 0.962. However, it was observed that the training set continued to decrease to MSE = 4.1 × 10^−3^ and R^2^ = 0.935, while the test set increased to MSE = 1.1 × 10^−2^ and R^2^ = 0.977. Therefore, a neuron with 20 hidden layers achieved the best results.

### 3.3. Optimal Model Results

**Figure 7:**
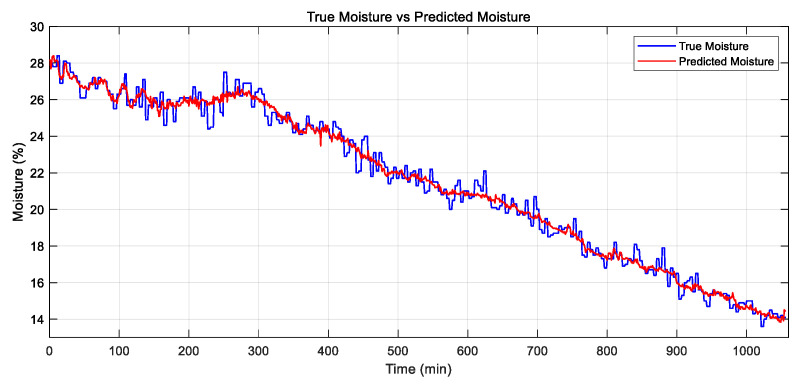
Comparison of predicted and actual values of the model.

Based on the results from the previous sections, the optimal neural network structure consists of a single hidden layer with 20 neurons, using a log-sigmoid (logsig) activation function in the hidden layer, a positive linear (poslin) transfer function in the output layer, and the Bayesian Regularization BP (br) for training, with 5-fold cross-validation. This network has 11 input features: time, initial moisture content, ambient temperature, drying temperature, drying humidity, exhaust temperature, exhaust humidity, grain temperature, airspeed, active power, and active energy. As previously noted, the model achieved the following performance: training and testing MSE values of 4.5 × 10^−3^ and 6.6 × 10^−3^ (R^2^ = 0.975 and 0.962), with 20 neurons and an optimal epoch count of 759. The predicted values of paddy drying closely matched the actual values, as shown in Figure 7, with no signs of overfitting.

## 4. Model Structure Optimization

The numerous inputs of the neural network increase its complexity. Moreover, the abundance of input variables contributes to the hardware complexity of the device, and interference from the drying environment, along with data distortion during remote transmission, is a noteworthy challenge. Therefore, simplifying the model structure while maintaining performance is crucial for practical applications.

### 4.1. Contribution Analysis of Drying Features

**Figure 8:**
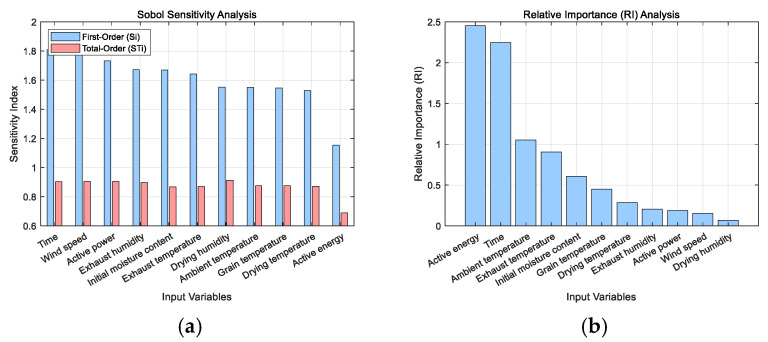
Input contribution based on Sobol sensitivity analysis and RI analysis: (**a**) Sobol sensitivity analysis; (**b**) RI analysis.

Figure 8 illustrates the impact of each input on the moisture content. The RI analysis indicates that the primary influential inputs on moisture content are time, initial moisture content, drying temperature, active energy, and ambient temperature. According to the first-order sensitivity index Si in the Sobol sensitivity analysis, the inputs that have an impact on moisture content, ranked in descending order, are time, airspeed, active power, initial moisture content, exhaust humidity, exhaust temperature, ambient temperature, drying humidity, drying temperature, grain temperature, and active energy. The total sensitivity indices of inputs, except for active energy, are relatively similar, indicating a balanced influence of each input variable and their interaction effects on the output.

This study employed two analytical approaches—Relative Importance (RI) analysis and Sobol global sensitivity analysis—to systematically evaluate the influence of each input variable on moisture content prediction. These methods assess input contributions from two complementary perspectives: RI reflects the direct pathway contribution of each variable within the neural network structure based on internal weight distributions, while the Sobol method quantifies the variance contribution of each input, including nonlinear interaction effects. Both methods demonstrated high consistency in identifying the primary influential variables, such as time and initial moisture content, thereby confirming the reliability of the model’s core inputs. However, discrepancies were observed for certain inputs—such as active energy—which showed high importance in RI analysis but low total effect in Sobol analysis. This divergence highlights the potential bias that may arise when relying on a single sensitivity analysis technique. By combining the results of both methods, the dual-perspective approach enables a more accurate identification of key variables while accounting for interaction effects, thereby reducing the risk of misjudgment. This integrated strategy not only preserves the integrity of input features but also facilitates effective network simplification and optimization, ultimately enhancing the interpretability and adaptability of the prediction model.

### 4.2. Model Optimization

To improve the model’s performance across all features, the inputs most influential on the moisture content were selected based on RI analysis and Sobol sensitivity analysis to construct the neural network. Additionally, neural network structures from other studies on moisture prediction for dryers were applied to the current equipment for comparison: based on RI sensitivity analysis, selecting time, active energy, initial moisture content, ambient temperature, and drying temperature as inputs (M1); based on Sobol sensitivity analysis, selecting time, initial moisture content, airspeed, active power, and drying humidity as inputs (M2); based on the input device performance parameters, selecting time, initial moisture content, air velocity, active power, and active energy as inputs (M3) [31]; based on temperature variations, using time, initial moisture content, ambient temperature, exhaust temperature, drying temperature, and grain temperature as inputs (M4) [20,23]; based on input and output temperature–humidity relationships, using time, initial moisture content, drying temperature, drying humidity, exhaust temperature, and exhaust humidity as inputs (M5) [21,22]; and using all inputs as one dataset (Mall).

Table 4 shows that the M4 model was found to be the best with training and testing MSE and R^2^ values of 4.8 × 10^−3^ and 0.973 and 6 × 10^−3^ and 0.967, respectively. Similar conclusions were drawn in studies on continuous grain dryers, indicating that predicting moisture content based on temperature variation within the dryer demonstrates good performance [20]. The poor performance of Model M5 may be attributed to differences in dryer types [21,22], suggesting that this neural network structure is not well suited for application to heat pump dryers. The testing R^2^ of M3 was the lowest (0.948), possibly because the referenced study employed microwave vacuum drying, where microwave power was the sole influencing factor. In contrast, for heat pump dryers, other factors affecting moisture content cannot be ignored [31]. M1 followed a similar trend with training and testing MSE and R^2^ values of 5.6 × 10^−3^ and 0.969 and 6.3 × 10^−3^ and 0.966, respectively. M2, M3, and M5 all performed less well than Mall. However, M1 and M4 required 14 and 20 neurons, with 5 and 6 input nodes, respectively. M4 marginally outperformed M1, but the M1 model was structurally simpler. Therefore, the M1 model (14 neurons, 5 inputs) was chosen as the best model, resulting in a substantially simpler structure than the Mall model (20 neurons, 11 inputs).

### 4.3. Comparison of Optimized Model and Mathematical Models

The optimized (M1) model outperformed empirical models in Table 5, although Wang and Singh’s model outperformed other empirical models with MSE ranging from 0.469 to 1.127 and R^2^ from 0.792 to 0.974. However, this is still lower than the M1 model’s performance, with MSE at 6.2 × 10^−3^ and R^2^ at 0.966. The Wang and Singh and Two term exponential models achieved relatively higher R^2^ values (0.974 and 0.970) in various drying tests. This is due to the steady state of elements such as hot air temperature and humidity, ambient circumstances, and airspeed causing the actual drying data to follow specific patterns. However, differences in moisture content and environmental conditions caused their performance not to be optimal. Significant changes in these components resulted in MSE values of 2.131 and R^2^ values as low as 0.571. This highlights the limitations of empirical models in describing the complex conditions of industrial-scale drying processes. The optimized (M1) model required only 36.284 s for the complete training process, demonstrating excellent training efficiency and strong scalability for handling larger datasets in the future. The prediction time for a single sample was just 0.0188 s, which fully meets the real-time prediction requirements of industrial drying processes.

### 4.4. Final Results of the Optimized Model

**Figure 9:**
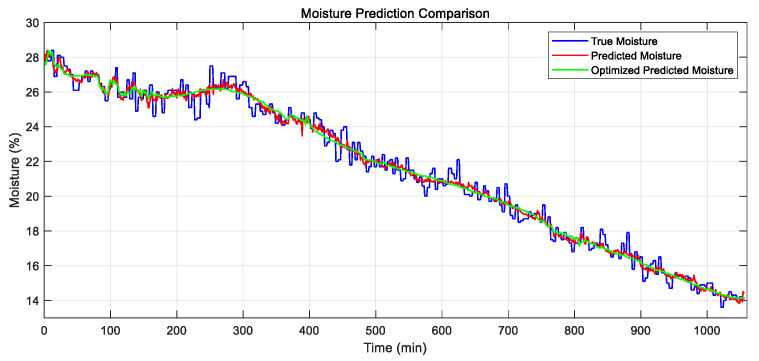
Comparison of moisture prediction values before and after optimization.

The RI analysis indicates that the optimized ANN model adopts a 5-14-1 topology, which represents a significant simplification compared to the original 11-20-1 structure. The log-sigmoid (logsig) function was used as the activation function in the hidden layer, and the positive linear (poslin) function was applied in the output layer. The model inputs included time, active energy, initial moisture content, ambient temperature, and drying temperature, and were trained using five-fold cross-validation. Figure 9 presents the predicted moisture values. There is no significant difference between the predictions of the optimized and non-optimized models.

**Figure 10:**
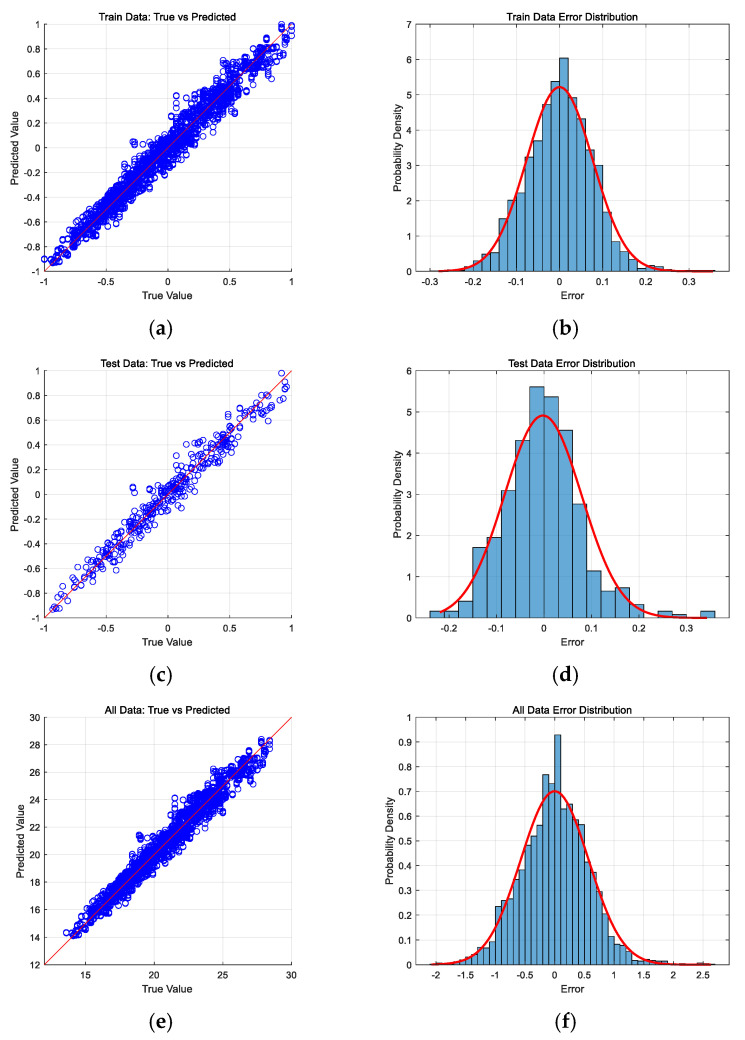
Comparison of actual values and error values for training, testing, and all datasets, and error distribution frequency histogram: (**a**) training comparison; (**b**) training error distribution; (**c**) testing comparison; (**d**) testing error distribution; (**e**) all comparison; and (**f**) all error distribution.

Figure 10 illustrates the prediction results and error distribution frequency histograms for the training set, test set, and entire dataset. The predicted values for each dataset align closely along the 45-degree line, while the residual errors exhibit a normal distribution. Table 6 shows the statistical characteristics of the prediction data. It was observed that there are minimal differences in the mean, variance, and overall values across the datasets which are negligible for practical applications.

The results were analyzed using the *t*-test, F-test, and Kolmogorov–Smirnov test to compare the means, variances, and distributions, respectively. The *p* values calculated for all three phases (training, testing, and entire dataset) are shown in Table 7. Results indicate high reliability as the *p* values at all stages are greater than 0.05. There are no significant differences in the mean, variance, or statistical distribution between the optimized model’s predictions and actual values.

## 5. Conclusions

This study aimed to predict the moisture content of paddy during drying in a low-temperature heat pump dryer. The drying characteristics were determined using an automated data collection scheme. These parameters include drying time, initial moisture content, ambient temperature, drying temperature, drying humidity, exhaust temperature, exhaust humidity, grain temperature, air velocity, active power, and active energy. An artificial neural network (ANN) model was developed to predict the paddy moisture content during drying. However, K-fold cross-validation was used to prevent overfitting. The optimal ANN model structure consisted of one hidden layer with 20 neurons utilizing a logistic sigmoid function for the hidden layer and a linear function for the output layer trained with Bayesian Regularization BP (br). The model structure was optimized to reduce and improve predictive performance. The results demonstrated that RI sensitivity analysis provided effective model optimization, yielding a simplified 5-14-1 topology with training and testing MSE values of 5.6 × 10^−3^ and 6.2 × 10^−3^ (R^2^ = 0.969 and 0.966). The residual errors conformed to a normal distribution, with *p* values exceeding 0.05, indicating no significant difference between the predicted and actual values. Future research will aim to investigate the quality of dried materials in heat pump dryers, exploring the relationships between material quality at different moisture levels and factors such as hot air temperature and airflow velocity. The predictive model developed in this study will be applied to control systems to enable early prediction of quality changes in materials, allowing for timely adjustments. This approach can effectively enhance drying quality at the industrial scale and increase economic benefits.

## Figures and Tables

**Table 1:** Sensor specifications.

Sensor Name	Model	Accuracy	Measurement Target
Temperature and humidity sensor	CWS-21-X-A1-G0 (Xingyi Sensor Manufacturing Co., Ltd., Langfang, China)	±0.1 °C/±1.5% RH	Drying and exhaust air temperature, humidity
Temperature sensor	CWDZ11-01-DZ-44(Xingyi Sensor Manufacturing Co., Ltd., Langfang, China)	±0.1 °C	Grain and ambient temperature
Anemometer	JY-GD680 (Zhongyi Liankong Technology Co., Ltd., Beijing, China)	0.2 m/s	Air velocity
Moisture analyzer	CSTIIC (Shizuoka Seiki Co., Ltd., Shizuoka, Japan)	±0.5%	Grain moisture content
Power meter	DTSD1946-L (Sfere Electric Co., Ltd., Jiangsu, China)	±1%	Active power and energy consumption of heat pump

**Table 2:** Transfer functions and mathematical definitions.

Transfer Function Name	MATLAB(R2022a) Symbol	Mathematical Formula
Sigmoid	logsig	$f(x)=\frac{1}{1+\exp(-x)}$
Hyperbolic tangent sigmoid	tansig	$f(x)=\frac{2}{1+\exp(-2x)}-1$
Elliot sigmoid	elliotsig	$f(x)=\frac{x}{1+|x|}$
Positive linear	poselin	$f(x)=\left\{\begin{array}{ll} x & \mathrm{if}\;x\geq 0\\ 0 & \mathrm{if}\;x<0 \end{array}\right.$
Radial basis	radbas	$f(x)=\exp(-x^{2})$
Triangular basis	tribas	$f(x)=\left\{\begin{array}{ll} 1-\mathrm{abs}(x), & \mathrm{if}\;-1\leq x\leq 1\\ 0, & \mathrm{otherwise} \end{array}\right.$

**Table 3:** Semi-empirical models for paddy drying.

Model Name	Mathematical Formula
Wang and Singh [27]	$MR=1+a\cdot t+b\cdot t^{2}$
Henderson and Pabis [27]	$MR=a\cdot e^{-k\cdot t}$
Two term exponential [28]	$MR=a\cdot e^{-k_{1}t}+(1-a)\cdot e^{-k_{2}t}$
Diffusion approach [29]	$MR=a\cdot e^{-kt}+b$
Midilli [30]	$MR=a\cdot e^{-k\cdot t^{n}}+b\cdot t$

**Table 4:** Optimal results of different model optimization methods.

Name	Training MSE	Training R^2^	Validation MSE	Validation R^2^	Test MSE	Test R^2^	Neurons	Inputs
M1	5.684 × 10^−3^	0.969	6.653 × 10^−3^	0.962	6.293 × 10^−3^	0.966	14	5
M2	6.575 × 10^−3^	0.964	7.807 × 10^−3^	0.955	8.474 × 10^−3^	0.954	9	5
M3	5.762 × 10^−3^	0.969	6.731 × 10^−3^	0.962	9.465 × 10^−3^	0.948	17	5
M4	4.878 × 10^−3^	0.973	6.163 × 10^−3^	0.965	6.029 × 10^−3^	0.967	20	6
M5	5.086 × 10^−3^	0.972	6.538 × 10^−3^	0.963	6.895 × 10^−3^	0.962	19	6
Mall	4.538 × 10^−3^	0.975	1.011× 10^−2^	0.944	6.634 × 10^−3^	0.962	20	11

**Table 5:** Comparison of optimized model and empirical models.

Name	MSE	R^2^
M1	6.293 × 10^−3^	0.966
Wang and Singh	0.469–1.127	0.792–0.974
Henderson and Pabis	0.353–1.967	0.761–0.937
Two term exponential	0.457–1.366	0.644–0.970
Diffusion approach	0.646–1.746	0.735–0.901
Midilli	0.571–2.131	0.571–0.922

**Table 6:** Statistical analysis of prediction results for training, testing, and all datasets.

Name		Mean	Variance	Standard Deviation	Minimum	Maximum	Kurtosis	Skewness
Training	Actual	−0.018	0.183	0.428	−1.000	1.000	2.248	0.079
	Predicted	−0.018	0.178	0.422	−0.932	0.999	2.208	0.061
Test	Actual	0.013	0.175	0.419	−0.932	0.959	2.444	0.051
	Predicted	0.011	0.166	0.407	−0.931	0.980	2.398	−0.026
All	Actual	20.889	9.997	3.162	13.600	28.400	2.266	0.076
	Predicted	20.888	9.691	3.113	14.100	28.392	2.224	0.052

**Table 7:** Hypothesis test results for training set, testing set, and all datasets.

Name	T Statistic	F Statistic	KS Statistic
All	0.989	0.319	0.062
Training	0.940	0.573	0.761
Test	0.992	0.387	0.074

## Data Availability

The raw data supporting the conclusions of this article will be made available by the authors on request.
